# Effects of selenium supplementation on pregnancy outcome and disease progression in HIV-infected pregnant women in Lagos, Nigeria: Study protocol for a randomised, double-blind, placebo-controlled trial

**DOI:** 10.1097/MD.0000000000012735

**Published:** 2019-01-18

**Authors:** Kehinde S. Okunade, Sarah John-Olabode, Oluwatosin J. Akinsola, Opeyemi Akinajo, Sulaimon A. Akanmu, Phyllis J. Kanki

**Affiliations:** a Department of Obstetrics & Gynaecology; b Department of Haematology & Blood Transfusion; c Epidemiology and Biostatistics unit, Department of Community Health & Primary Health Care, College of Medicine, University of Lagos; d Department of Obstetrics & Gynaecology, Lagos University Teaching Hospital, Nigeria; e Department of Immunology & Infectious Diseases, Harvard School of Public Health, USA.

**Keywords:** CD4+ cells count, lagos, low birth weight, LUTH, preterm birth, selenium, viral load

## Abstract

**Background:**

Micronutrient deficiencies are common during pregnancy, especially in pregnant women from economically disadvantaged settings where diets with low content of minerals and vitamins are consumed. Selenium is a non-metallic chemical element of great importance to human health. This study will assess the effect of selenium supplementation on major pregnancy outcomes and disease progression among HIV-infected pregnant women in Lagos, Nigeria.

**Methods:**

A randomized, double-blind, placebo-controlled trial involving confirmed HIV-positive pregnant women at the Lagos University Teaching Hospital (LUTH) between September 2018 and February 2019. Eligible participants are HIV-infected pregnant women aged 15 to 49 years and have a singleton gestation at 14 to 27 weeks’ gestation. At enrolment, 90 women will be randomly assigned into each intervention arm to receive either a daily tablet of 200 μg elemental selenium or placebo. Relevant participants’ data will be collected at enrolment and at delivery. Statistical analyses will be carried out using SPSS version 23.0 for Windows. The associations between any 2 groups of continuous variables will be tested using the *t* test or the Mann-Whitney *U* test and that of 2 groups of categorical variables with chi-square or Fishers exact test where appropriate. A series of multivariable analyses will also be carried out to identify and control for several possible confounders of the major pregnancy outcomes and HIV disease progression. Statistical significance will be defined as *P* < .05. Ethical approval for the study was obtained from the LUTH's Health Research and Ethics Committee (Approval number: ADM/DCST/HREC/APP/2438; 30th August 2018).

**Discussion:**

This trial will assess the effect of selenium supplementation on pregnancy outcome and HIV disease progression among HIV-infected pregnant women in Lagos. This will help to determine if routine selenium supplementation in HIV-infected pregnant women will contribute to the improvement in the major adverse pregnancy outcomes such as preterm birth and low birth weight and the HIV disease surrogate markers such as CD4+ cells count and viral load.

**Trial registration:**

PACTR, PACTR201809756724274. Registered on 3rd September 2018, https://pactr.samrc.ac.za/TrialDisplay.aspx?TrialID=3571.

## Abstract 2

Supplemental Digital Content is available in the text

## 1 Introduction

Micronutrient deficiencies are common during pregnancy, especially in pregnant women from economically disadvantaged settings where diets with low content of minerals and vitamins are consumed.^[1]^ Selenium is a non-metallic chemical element of great importance to human health.^[2,3]^ It is a key component of several human seleno-proteins and mostly involved in redox reactions.^[4]^ It acts as an antioxidant and helps protect the body against the damaging effects of free radicals.^[5]^ Selenium is essential for the activity of the enzyme glutathione peroxidase (GPX), a main intracellular antioxidant that protects against reactive oxygen species and subsequent oxidation-induced cellular damage.^[5,6]^ Biochemical selenium deficiency has been associated with increased mortality among those infected with HIV^[7]^ and with accelerated HIV disease progression through increased viral load.^[8]^ Selenium's role in antioxidant defence^[9]^ and immunity^[10]^ may be the underlying mechanism. There is, however, limited data on the selenium status of Nigerian pregnant women with HIV-infection, with a recent study conducted by us showing a prevalence rate of selenium deficiency to be 20.4% and we reported significantly increased rates of preterm birth and delivery low birth weight babies among the HIV infected participants with selenium deficiency.^[11]^ Several other studies have also reported low selenium status in HIV-infected individuals, and that serum selenium concentration declines with disease progression.^[12,13]^

Selenium is found in cereals, meat, poultry, seafood, and eggs.^[6]^ Cereals may provide about 50% of dietary selenium, however data on the selenium content of Nigerian or African foods is also limited. The selenium content of plant foods varies with the selenium content of the soil.^[6]^ The Recommended Dietary Intake of selenium for pregnant women is 60 mg/day^[6]^ and most interventional studies make use of daily doses of up to 200 μg selenium in its elemental form (selenomethionine).^[14–17]^ Supplemental doses up to 200 μg of the mineral should not cause adverse effects, even if temporarily exceeded, since the selenium upper tolerable intake (UL) for this group is established as 400 μg (5.1 μmol) selenium/day.^[18]^ Given the high UL and the regulation of body homeostasis through urinary excretion, selenium supplementation can be regarded as a safe intervention.^[18,19]^ There is still limited data about selenium toxicity in humans, but the most common side effects include: hair and nail brittleness and loss, gastrointestinal disturbances, skin rash, fatigue, irritability and nervous system disturbances.^[6,19]^ Selenium containing supplements are not recommended for children under the age of 15 years.^[6]^ There is currently paucity of studies that examined the effects of selenium supplementation among pregnant HIV-infected black African women. This study will therefore attempt to assess the effect of antenatal selenium supplementation on major pregnancy outcomes (preterm birth and low birth weight) and HIV disease progression rate among pregnant women at the Lagos University Teaching Hospital (LUTH), Lagos, Nigeria.

## 2 Methods and design

### 2.1 Study design and setting

The study is designed as a prospective, randomized, double-blind, placebo-controlled, single-center trial involving confirmed HIV-seropositive pregnant women who are seeking care at the antenatal clinics of the Lagos University Teaching Hospital (LUTH). LUTH is the teaching hospital of the College of Medicine, University of Lagos. It has more than 1000 beds and is located in the metropolis of Lagos in Southwest Nigeria. The hospital provides services to patients from the neighbouring states in Southwest Nigeria. It is the largest hospital in Lagos State and mainly offers clinical services, including prenatal, intrapartum, and postnatal care. Pregnant women with HIV infections are jointly managed by haematologists and HIV physicians at the hospital's AIDS Prevention Initiative Nigeria (APIN) clinic and undergo regular monitoring of their disease with 3-monthly CD4+ cells count and viral load. Participants’ enrolment will start in September 2018, and the last woman is expected to be included in the trial in February 2019. The Health Research and Ethics Committee of LUTH's Institutional Review Board has granted ethics approval for this study (Approval Number ADM/DCST/HREC/APP/2438; 30th August 2018).

### 2.2 Study population/ participant enrolment

Eligible participants are confirmed HIV-seropositive pregnant women aged 15 to 49 years who have a singleton gestation at 14 to 27 weeks’ gestation. The exclusion criteria at enrolment are multiple gestations, significant renal and hepatic impairment, an expected delivery date beyond March 2019, receipt of a long course of mineral supplement containing selenium during the 6 months prior to enrolment, refusal of consent at enrolment or withdrawal of consent during the study.

### 2.3 Randomisation and blinding schedule

Eligible women will be invited to give consent for participation in the trial within 2 weeks of screening. At enrolment, participants will be randomly assigned by a trained research assistant into 2 arms to receive daily oral tablet of 200 μg elemental selenium (arm I) or placebo (arm II) till their delivery using a 1:1 block randomisation code to be generated from Random Allocation software version 1.0 (May 2004) by the study Statistician. The proposed dose of selenium is one-half of the safe and tolerable upper intake level of 400 μg/day for pregnant women.^[18]^ The active drug (selenium) and placebo tablets will be indistinguishable in shape, size, and colour and will be packed in identical coded transparent dispensing sachets by the onsite pharmacy technician who will then store the coded randomization list in a sealed opaque envelope that will be kept in a locked file cabinet at the study site until the end of the study. Twelve (12) dispensing sachets with identical numeric regimen code, containing the same intervention drug will be prepared for each of the participants. At every 2-weekly follow-up visit, a new dispensing sachet with the same numeric code, containing 16 tablets of the same intervention drugs will be given to each woman and pills remaining in used sachets will be counted to assess compliance. In addition, all the women will receive the standard prenatal care (see below). Study investigators and participants will be blinded to the treatment arms until after the final analysis. The onsite pharmacy technician is not involved in the design, conduct, statistical analysis, and reporting of study findings.

### 2.4 Standard prenatal care

All the women will receive their routine prenatal care which includes daily doses of ferrous gluconate (300 mg, equivalent to 60 mg of elemental iron) and folic acid (5 mg), and 4-weekly doses of malarial chemoprophylaxis in the form of Sulfadoxine-Pyrimethamine (SP) tablets (from enrolment to 36 weeks of gestation) or instead daily doses of Cotrimoxazole 980 mg for women with CD4 + cell counts < 200 cells/mm^3^ according to established protocol for *Pneumocystis carinii* prophylaxis^[20]^ irrespective of treatment assignments. Alternatively, daily proguanil tablet (100 mg) is given to women with known or recent allergy to Sulphonamides from enrolment to delivery. All women will also be commenced on ART at some point during their follow-up according to the National HIV prevention and treatment guidelines.^[21]^

### 2.5 General procedure and monitoring

#### 2.5.1 Data collection and management

**Figure 1:**
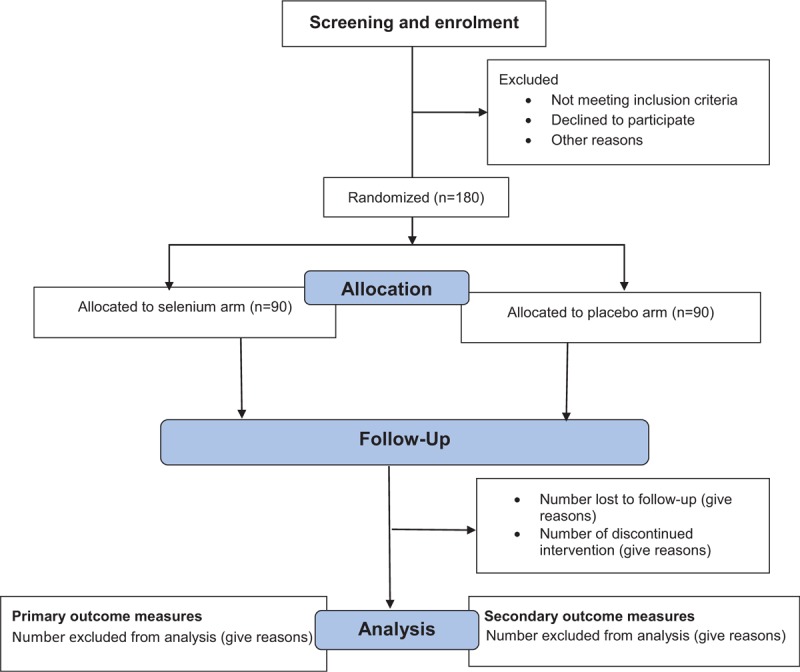
Trial flow chart.

At enrolment, the investigators will collect data on the participants’ socioeconomic status, education, and obstetric history, duration of HIV diagnosis (in months), WHO clinical stage of the disease,^[22]^ type and duration of ART use (in months), baseline Haemoglobin (Hb) levels (in g/dL), CD4 + cells count (in cells/mm^3^) and viral load (in copies/mL), and BMI (actual maternal weight is calculated by subtracting the estimated fetal weight at enrolment using a normogram chart from the measured maternal weight) (in kg/m^2^) using a structured interviewer-administered questionnaire. Gestational age is based on the date of the participant's last menstrual period, which will be obtained at the time of random assignment. Data collection begins on the day a participant is allocated into an intervention arm and continues until the termination of the trial or until the participant withdraws from the trial at any time for any reason. At delivery, data will be collected from the participants’ case notes on the latest CD4 + cells count and viral load (done within the last 6 weeks of delivery), WHO stage of the disease, and latest Hb levels. The total duration of supplement intake – selenium or placebo (in weeks), level of compliance to use of the supplement (good compliance level is defined as usage of at least 10 of the 16, 2-weekly prescribed tablets), mode of delivery, gestational age at delivery (in weeks) and the baby's birth weight (in grams) will also be recorded in the questionnaire. Original data will be transferred to an electronic database system located in a guarded facility at the trial site by the research assistant. Access to the study data is restricted. The PI will have the access to the final dataset. An independent steering committee will monitor and examine strict compliance with the study protocol (Fig. 1 and Table 1). Trial progression or protocol modification will be updated in the registered database (Pan African Clinical Trial Registry, PACTR) and reported to the LUTH's ethical committee that will provide the oversight functions of data and safety monitoring in the course of the study.

**Table 1:**
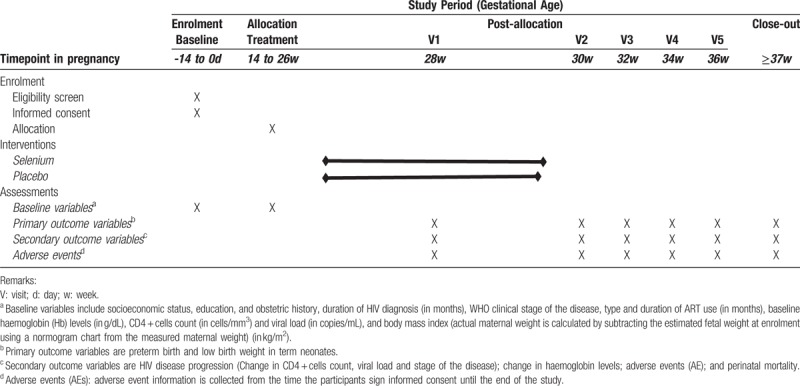
Schedule of enrolment, interventions, and outcome assessments in the trial.

### 2.6 Confirmatory outcome variables

Primary endpoints – Preterm birth (delivery before 37 completed weeks of pregnancy) and low birth weight in term neonates (baby's birth weight less than 2.5 kg).

Secondary endpoints – HIV disease progression (change in CD4 + cells count, viral load and stage of the disease); mid-trimester miscarriage, change in haemoglobin levels; adverse events (AE) such as hepatotoxicity, gastrointestinal disturbances, dermatologic effects (nail and hair loss and dermatitis), and neurotoxicity; and perinatal mortality (number of stillbirths and neonatal deaths in the first week of life).

### 2.7 Statistical methods

#### 2.7.1 Sample size

Using the data from our previous study,^[11]^ we propose to enrol 148 participants in the study to investigate the effect of selenium supplementation on the primary endpoints. The study is powered for a 2-sided test with a Type I error rate of 5% and 95% power, that is, Z_α_ = 1.96 and Z_β_ = 1.64, adjusted for a 20% drop-out rate. We also propose to enrol 180 participants to detect an effect size of 0.3 of selenium supplementation on the major secondary endpoint (reduction in HIV disease progression). This is powered for a 2-sided test with a Type I error rate of 5% and 80% power, that is, Z_α_ = 1.96 and Z_β_ = 1.64, adjusted for a 20% drop-out rate. Therefore, a total sample size of 180 is used in this study with 90 HIV-infected women randomised into each of the trial arms to assess the effects of selenium supplementation on the primary and secondary outcome variables.

### 2.8 Statistical analyses

The intention-to-treat principle will be used in the final analyses. Statistical analyses will be carried out using SPSS version 23.0 for Windows (Armonk, NY: IBM Corp.). The associations between any 2 groups of continuous variables will be tested using the independent sample or paired *t* test (normal distribution) or the Mann-Whitney *U* test (skewed data) and that of 2 groups of categorical variables with chi-square or Fishers exact test where appropriate. A series of multivariable analyses using binary logistic regression model will also be carried out to identify and control for several possible confounders of the primary and secondary outcome variables. Statistical significance will be defined as *P* < .05.

### 2.9 Discontinuation of study intervention (code-breaking) and Reporting

The PI may choose to discontinue a participant from the study for reasons such significant failure to compliance with study intervention, serious adverse event (SAE) such as significant renal or hepatic impairment or suspected unexpected serious adverse reaction (SUSAR), that is, medical condition or situation in which continued participation in the study would not be in the best interest of the participant (code-breaking). The event causing study intervention discontinuation will then be documented in a dedicated Case Report Form (CRF) that would capture the date and the specific underlying reason for discontinuation of study intervention.

### 2.10 Quality control and data monitoring

All investigators, research assistant and onsite pharmacy technician will be required to undergo training prior to the trial to guarantee consistent practice. The training will include understanding of inclusion/exclusion/exit criteria, follow-up procedures, and completion of questionnaire and CRF. The trial will be monitored by quality assurance personnel from the research management office of the College of Medicine, University of Lagos, who will be independent from the study team, and an independent steering committee. There will be a periodic monitoring to guarantee accuracy and quality throughout the study period. The essential documents (consent information, enrolment, protocol deviations, number and proportion of missed visits, and losses to follow-up) will be monitored and checked for accuracy and completeness by the monitors. The principal investigator (PI) is responsible for the overall project and for organizing steering committee meetings. An independent steering committee will be responsible for ensuring the overall safety of participants, coordinating study meetings, supervising the study, monitoring data safety, and overseeing quality control.

### 2.11 Ethics and dissemination

The study protocol was approved by the Health Research and Ethics Committee of the Lagos University Teaching Hospital on September 30th, 2018 with approval number ADM/DCST/HREC/APP/2438 [Additional file 1]. Participants will provide written informed consent [Additional file 2] prior to participation in the trial and the participant will keep a signed copy of the consent form for future references. Participants below the age of 18 years will be regarded as emancipated minors. ‘Emancipated minor’ is a person that is not of legal age to give consent (below 18 years of age in Nigeria) for a research study but who by marriage, pregnancy, being the mother of a child whether married or not or has left home and is self-sufficient can be allowed to do so legally. The trial will be reported in line with the Standard Protocol Items: Recommendations for Interventional Trials (SPIRIT) checklist [Additional file 3] and the results will be disseminated in peer review scientific journals.

## 3 Discussion

Selenium is a non-metallic chemical element of great important to human health. Biochemical selenium deficiency has been associated with increased mortality among those infected with HIV and with accelerated HIV disease progression. This study aims to assess the effects of giving routine antenatal selenium supplementation to HIV-infected pregnant women on their pregnancy outcome and HIV-disease progression. If it is found to be significantly effective and safe, this trial will provide a basis for prescribing this antioxidant to HIV-seropositive women in pregnancy.

### 3.1 Trial status

At the time of initial manuscript submission, recruitment is yet to commence into the trial. Participants’ enrolment will start in September 2018, and the last woman is expected to be included in the trial in February 2019. The manuscript reports protocol version 2.0 (30th June 2018).

## Acknowledgments

The authors would like to thank the HBNU Global Health Fellows Consortium for providing the funding for this work. We also appreciate the efforts of Prof. Folasade Ogunsola (Deputy Vice-Chancellor, Development Services of the University of Lagos, Lagos, Nigeria) in making the application for the HBNU Fellowship a success through her very valuable criticisms.

## Author contributions

KSO, SAA, and PJK participated in the conception, design, and writing of the study protocol. SJ-O contributed to the revision and editing of the study protocol. KSO, SJ-O, OJA and OA will be involved in the recruitment of participants and the acquisition of data. KS wrote the first draft of this manuscript. SJ-O, OJA, OA, SA, PK were all involved in the revision of the manuscript. All authors approved the final version of the manuscript to be submitted.

**Conceptualization:** Kehinde Sharafadeen Okunade, Sulaimon A Akanmu, Phyllis J Kanki.

**Formal analysis:** Oluwatosin J Akinsola.

**Funding acquisition:** Kehinde Sharafadeen Okunade, Sulaimon A Akanmu, Phyllis J Kanki.

**Methodology:** Kehinde Sharafadeen Okunade, Sarah John-Olabode, Oluwatosin J Akinsola, Opeyemi Akinajo, Sulaimon A Akanmu, Phyllis J Kanki.

**Project administration:** Kehinde Sharafadeen Okunade.

**Resources:** Kehinde Sharafadeen Okunade.

**Software:** Kehinde Sharafadeen Okunade, Oluwatosin J Akinsola.

**Supervision:** Kehinde Sharafadeen Okunade, Sarah John-Olabode, Opeyemi Akinajo, Sulaimon A Akanmu.

**Validation:** Kehinde Sharafadeen Okunade.

**Visualization:** Kehinde Sharafadeen Okunade.

**Writing – original draft:** Kehinde Sharafadeen Okunade, Sarah John-Olabode, Opeyemi Akinajo.

**Writing – review & editing:** Kehinde Sharafadeen Okunade, Sarah John-Olabode, Oluwatosin J Akinsola, Opeyemi Akinajo, Sulaimon A Akanmu, Phyllis J Kanki.

Kehinde Sharafadeen Okunade orcid: 0000-0002-0957-7389.

## Supplementary Material

Supplemental Digital Content
