# Supplementary material for: Effects of selenium supplementation on pregnancy outcome and disease progression in HIV-infected pregnant women in Lagos, Nigeria: Study protocol for a randomised, double-blind, placebo-controlled trial
Source: Medicine (Baltimore). 2019 Jan 18;98(3):e12735. doi: 10.1097/MD.0000000000012735 (PMC6370178; doi:10.1097/MD.0000000000012735)
Supplement: Supplemental Digital Content [file medi-98-e12735-s001.pdf]

LAGOS UNIVERSITY TEACHING HOSPITAL
HEALTH RESEARCH ETHICS COMMITTEE

PRIVATE MAIL BAG 12003, LAGOS, NIGERIA
e-mail address: luthethics@yahoo.com

Chairman
PROF. N.U. OKUBADEJO
MB. ChB, FMCP

Administrative Secretary
D.J. AKPAN
B.Sc. (Hons) BUS. ADMIN,
MIHSAN

Chief Medical Director:
PROF. CHRIS BODE
FMCS (NIG) FWACS

Chairman, Medical Advisory Committee
PROF. O.A. FASANMADE
MBBS, FWACP, FACE, FNSEM

LUTH HREC REGISTRATION NUMBER: NHREC: 19/12/2008a
Office Address: Room 107, 1st Floor, LUTH Administrative Block
Telephone: 234-1-5850737, 5852187, 5852209, 5852158, 5852111

30th August, 2018

NOTICE OF EXPEDITED REVIEW AND APPROVAL

PROJECT TITLE: "A RANDOMISED, DOUBLE-BLIND, PLACEBO-CONTROLLED TRIAL OF THE EFFECTS OF SELENIUM SUPPLEMENTATION ON PREGNANCY OUTCOME AND DISEASE PROGRESSION IN HIV-INFECTED PREGNANT WOMEN IN LAGOS, NIGERIA".
HEALTH RESEARCH COMMITTEE ASSIGNED NO.: ADM/DCST/HREC/APP/2438
NAME OF PRINCIPAL INVESTIGATOR: DR. KEHINDE S. OKUNADE
ADDRESS OF PRINCIPAL INVESTIGATOR: DEPT. OF OBSTETRICS AND GYNAECOLOGY, LUTH.
DATE OF RECEIPT OF VALID APPLICATION: 30-07-18

This is to inform you that the research described in the submitted protocol, the consent forms, and all other related materials where relevant have been reviewed and given full approval by the Lagos University Teaching Hospital Health Research Ethics Committee (LUTHHREC).

This approval dates from 30-08-2018 to 30-08-2019. If there is delay in starting the research, please inform the HREC so that the dates of approval can be adjusted accordingly. Note that no participant accrual or activity related to this research may be conducted outside of this dates. All informed consent forms used in this study must carry the HREC assigned number and duration of HREC approval of the study. In multiyear research, endeavor to submit your annual report to the HREC early in order to obtain renewal of your approval and avoid disruption of your research.

The National code for Health Research Ethics requires you to comply with all institutional guidelines, rules and regulations and with the tenets of the code including ensuring that all adverse events are reported promptly to the HREC. No changes are permitted in the research without prior approval by the HREC except in circumstances outlined in the code. The HREC reserves the right to conduct compliance visits to your research site without previous notification.

CHAIRMAN
HEALTH RESEARCH & ETHICS COMMITTEE
LUTH

PROF. N. U. OKUBADEJO
CHAIRMAN, LUTH HEALTH RESEARCH ETHICS COMMITTEE

CONSENT FORM

ADM/DCST/HREC/APP/2438 (30th August 2018 to 30th August 2019)

Title of Research: A Randomised, Double-Blind, Placebo-Controlled Trial of the Effects of Selenium Supplementation on Pregnancy Outcome and Disease Progression in HIV-infected Pregnant Women in Lagos, Nigeria.

Name & Affiliation of Researcher: This study is being conducted by Dr. K. S. Okunade of the Department of Obstetrics and Gynaecology and other researchers in the College of Medicine, University of Lagos/ Lagos University Teaching Hospital, Idi-Araba, Lagos.

Introduction: Lack of nutrition are common during pregnancy, especially in pregnant women from low resource settings where diets with low minerals and vitamins are consumed. Lack of selenium in the body has been associated with increased deaths among those infected with HIV and with worsening HIV disease. However, there are only few researches that have looked at the effect of giving selenium as supplement to HIV-infected pregnant women on their pregnancy outcome and if this will improve their HIV disease.

Purpose(s) of the research: We want to find out if giving selenium supplements to pregnant women will improve their pregnancy outcomes such as reducing the number of deliveries before 9 months and the number of babies born with low birth weight and if their HIV disease will be improved.

Procedure of the research: This study will involve HIV-infected pregnant women and they will be asked to participate in the study when they are 3 to 6 months pregnant between August 2018 and January 2019. At the time of joining the study, the women will be divided in equal numbers to receive either their regular antenatal drugs with a daily tablet of selenium or their regular antenatal drugs with another unknown vitamin. The researchers and the women who are participating in this study will not know what each woman in any of the 2 groups is taking.

Potential benefit(s) of the research: This study will give us the opportunity of knowing whether giving HIV-infected pregnant women selenium tablet in addition to their regular antenatal drugs will have effect on their pregnancy outcomes and if there will be improvement of their HIV disease. The results that we will get from the study will help us to know how to manage HIV-infected pregnant women better in the future. If the new treatment is proven to work and you're in the group getting it, you might be among the first to benefit. If you're in the group not getting the drug, you will still receive your regular antenatal care.

Potential risk(s): Women who take part in this study are not exposed to any serious risks. The new treatment being studied does not have any serious side-effects. However, the researcher may choose to stop any woman from further participation in the study if there is a report of any

serious side-effect or other medical condition or situation in which continued participation in the study would not be in the best interest of the woman. Discontinuation from study intervention does not mean discontinuation from the study, and remaining study procedures would still be completed as indicated by the study protocol.

Confidentiality: All the information we get from this study shall be kept strictly confidential. You are assured that your identity will be kept in confidence by the researchers and will never be revealed to another person.

Willingness to Participants: Your participation in this research is entirely voluntary and if you choose not to participate again, no punishment will be attached to your decision. You will not be paid any fees for participating in this research. You can choose to withdraw your participation in the research at any time.

What happens to research participants and the research setting when the research is over: The Researchers will display the results at the Gynaecology out-patient clinic and the Department of Obstetrics and Gynaecology for the patients and staff as part of medical education and use for patients care in the future.

Statement of person obtaining informed consent:

I have fully explained this research to the respondent and have given sufficient information, including the risks and benefits, to make an informed decision.

Date……..……..……………………… Signature………………………………….

Statement of person obtaining informed consent:

I have read the description of the research. I understand that any participation is voluntary. I know enough about the purpose, methods, risks and benefits of the research study to judge that I want to take part in it. I have received a copy of this consent form to keep for myself.

Date……..……..……………… Signature/Thumbprint…………………………

For further enquiry, please contact:

1. **Researcher's contact:**
   Dr. K. S. Okunade
   Department of Obstetrics & Gynaecology,
   College of Medicine, University of Lagos/Lagos University Teaching Hospital,
   Mobile: 08034728139
   Email: kehindeokunade@gmail.com

2. **LUTH Health Research & Ethics Committee's contact:**
   Room 107, Administrative block,
   Lagos University Teaching Hospital,
   Idi-Araba, Lagos

SPIRIT 2013 Checklist: Recommended items to address in a clinical trial protocol and related documents*

Section/item	Item No	Description	Addressed on page number
**Administrative information**			
Title	1	Descriptive title identifying the study design, population, interventions, and, if applicable, trial acronym	1
Trial registration	2a	Trial identifier and registry name. If not yet registered, name of intended registry	2
	2b	All items from the World Health Organization Trial Registration Data Set	
Protocol version	3	Date and version identifier	________11_____
Funding	4	Sources and types of financial, material, and other support	12
Roles and responsibilities	5a	Names, affiliations, and roles of protocol contributors	1
	5b	Name and contact information for the trial sponsor	_____________
	5c	Role of study sponsor and funders, if any, in study design; collection, management, analysis, and interpretation of data; writing of the report; and the decision to submit the report for publication, including whether they will have ultimate authority over any of these activities	
	5d	Composition, roles, and responsibilities of the coordinating centre, steering committee, endpoint adjudication committee, data management team, and other individuals or groups overseeing the trial, if applicable (see Item 21a for data monitoring committee)	9-10

**Introduction**			
Background and rationale	6a	Description of research question and justification for undertaking the trial, including summary of relevant studies (published and unpublished) examining benefits and harms for each intervention	1-2
	6b	Explanation for choice of comparators	________
Objectives	7	Specific objectives or hypotheses	4
Trial design	8	Description of trial design including type of trial (eg, parallel group, crossover, factorial, single group), allocation ratio, and framework (eg, superiority, equivalence, noninferiority, exploratory)	5-6
**Methods: Participants, interventions, and outcomes**			
Study setting	9	Description of study settings (eg, community clinic, academic hospital) and list of countries where data will be collected. Reference to where list of study sites can be obtained	5
Eligibility criteria	10	Inclusion and exclusion criteria for participants. If applicable, eligibility criteria for study centres and individuals who will perform the interventions (eg, surgeons, psychotherapists)	5-6
Interventions	11a	Interventions for each group with sufficient detail to allow replication, including how and when they will be administered	6-7
	11b	Criteria for discontinuing or modifying allocated interventions for a given trial participant (eg, drug dose change in response to harms, participant request, or improving/worsening disease)	9
	11c	Strategies to improve adherence to intervention protocols, and any procedures for monitoring adherence (eg, drug tablet return, laboratory tests)	7
	11d	Relevant concomitant care and interventions that are permitted or prohibited during the trial	6-7
Outcomes	12	Primary, secondary, and other outcomes, including the specific measurement variable (eg, systolic blood pressure), analysis metric (eg, change from baseline, final value, time to event), method of aggregation (eg, median, proportion), and time point for each outcome. Explanation of the clinical relevance of chosen efficacy and harm outcomes is strongly recommended	8
Participant timeline	13	Time schedule of enrolment, interventions (including any run-ins and washouts), assessments, and visits for participants. A schematic diagram is highly recommended (see Figure)	7-8

Sample size	14	Estimated number of participants needed to achieve study objectives and how it was determined, including clinical and statistical assumptions supporting any sample size calculations	8-9
Recruitment	15	Strategies for achieving adequate participant enrolment to reach target sample size	9

Methods: Assignment of interventions (for controlled trials)

Allocation:			
Sequence generation	16a	Method of generating the allocation sequence (eg, computer-generated random numbers), and list of any factors for stratification. To reduce predictability of a random sequence, details of any planned restriction (eg, blocking) should be provided in a separate document that is unavailable to those who enrol participants or assign interventions	6
Allocation concealment mechanism	16b	Mechanism of implementing the allocation sequence (eg, central telephone; sequentially numbered, opaque, sealed envelopes), describing any steps to conceal the sequence until interventions are assigned	6
Implementation	16c	Who will generate the allocation sequence, who will enrol participants, and who will assign participants to interventions	6
Blinding (masking)	17a	Who will be blinded after assignment to interventions (eg, trial participants, care providers, outcome assessors, data analysts), and how	6
	17b	If blinded, circumstances under which unblinding is permissible, and procedure for revealing a participant's allocated intervention during the trial	9

Methods: Data collection, management, and analysis

Data collection methods	18a	Plans for assessment and collection of outcome, baseline, and other trial data, including any related processes to promote data quality (eg, duplicate measurements, training of assessors) and a description of study instruments (eg, questionnaires, laboratory tests) along with their reliability and validity, if known. Reference to where data collection forms can be found, if not in the protocol	7
	18b	Plans to promote participant retention and complete follow-up, including list of any outcome data to be collected for participants who discontinue or deviate from intervention protocols	9

Data management	19	Plans for data entry, coding, security, and storage, including any related processes to promote data quality (eg, double data entry; range checks for data values). Reference to where details of data management procedures can be found, if not in the protocol	8
Statistical methods	20a	Statistical methods for analysing primary and secondary outcomes. Reference to where other details of the statistical analysis plan can be found, if not in the protocol	9
	20b	Methods for any additional analyses (eg, subgroup and adjusted analyses)	
	20c	Definition of analysis population relating to protocol non-adherence (eg, as randomised analysis), and any statistical methods to handle missing data (eg, multiple imputation)	
**Methods: Monitoring**			
Data monitoring	21a	Composition of data monitoring committee (DMC); summary of its role and reporting structure; statement of whether it is independent from the sponsor and competing interests; and reference to where further details about its charter can be found, if not in the protocol. Alternatively, an explanation of why a DMC is not needed	10
	21b	Description of any interim analyses and stopping guidelines, including who will have access to these interim results and make the final decision to terminate the trial	8
Harms	22	Plans for collecting, assessing, reporting, and managing solicited and spontaneously reported adverse events and other unintended effects of trial interventions or trial conduct	9
Auditing	23	Frequency and procedures for auditing trial conduct, if any, and whether the process will be independent from investigators and the sponsor	9-10
**Ethics and dissemination**			
Research ethics approval	24	Plans for seeking research ethics committee/institutional review board (REC/IRB) approval	10
Protocol amendments	25	Plans for communicating important protocol modifications (eg, changes to eligibility criteria, outcomes, analyses) to relevant parties (eg, investigators, REC/IRBs, trial participants, trial registries, journals, regulators)	9

Consent or assent	26a	Who will obtain informed consent or assent from potential trial participants or authorised surrogates, and how (see Item 32)	6
	26b	Additional consent provisions for collection and use of participant data and biological specimens in ancillary studies, if applicable	
Confidentiality	27	How personal information about potential and enrolled participants will be collected, shared, and maintained in order to protect confidentiality before, during, and after the trial	8
Declaration of interests	28	Financial and other competing interests for principal investigators for the overall trial and each study site	12
Access to data	29	Statement of who will have access to the final trial dataset, and disclosure of contractual agreements that limit such access for investigators	12
Ancillary and post-trial care	30	Provisions, if any, for ancillary and post-trial care, and for compensation to those who suffer harm from trial participation	
Dissemination policy	31a	Plans for investigators and sponsor to communicate trial results to participants, healthcare professionals, the public, and other relevant groups (eg, via publication, reporting in results databases, or other data sharing arrangements), including any publication restrictions	11
	31b	Authorship eligibility guidelines and any intended use of professional writers	
	31c	Plans, if any, for granting public access to the full protocol, participant-level dataset, and statistical code	12
**Appendices**			
Informed consent materials	32	Model consent form and other related documentation given to participants and authorised surrogates	
Biological specimens	33	Plans for collection, laboratory evaluation, and storage of biological specimens for genetic or molecular analysis in the current trial and for future use in ancillary studies, if applicable	

*It is strongly recommended that this checklist be read in conjunction with the SPIRIT 2013 Explanation & Elaboration for important clarification on the items. Amendments to the protocol should be tracked and dated. The SPIRIT checklist is copyrighted by the SPIRIT Group under the Creative Commons "Attribution-NonCommercial-NoDerivs 3.0 Unported" license.
